# Cannabis Laws and Opioid Use Among Commercially Insured Patients With Cancer Diagnoses

**DOI:** 10.1001/jamahealthforum.2025.3512

**Published:** 2025-10-17

**Authors:** Felipe Lozano-Rojas, Victoria Bethel, Sumedha Gupta, Shelby R. Steuart, W. David Bradford, Amanda J. Abraham

**Affiliations:** 1 Department of Public Administration and Policy, University of Georgia, Athens; 2 Department of Economics, Indiana University, Indianapolis; 3 Crown Family School of Social Work, Policy, and Practice, University of Chicago, Chicago, Illinois

## Abstract

**Importance:**

Pain is a prevalent cancer-related symptom, but limited research investigates whether cannabis is an effective analgesic for cancer pain.

**Objective:**

To examine the association of medical and recreational cannabis dispensary availability on prescription opioid dispensing among commercially insured patients with cancer.

**Design, Setting, and Participants:**

This cross-sectional study used synthetic control to investigate the association of cannabis dispensary openings with pain medication dispensing among patients with cancer. Data were extracted from Optum’s deidentified Clinformatics Data Mart database from January 1, 2007, to December 31, 2020. The study population included patients aged 18 to 64 years with a cancer diagnosis and at least 6 months of continuous enrollment. Associations were estimated by age, race and ethnicity, and sex. Data were analyzed between December 2024 and February 2025.

**Exposures:**

Exposures included indicators for whether a medical or recreational cannabis dispensary was open in each state-quarter.

**Main Outcomes and Measures:**

The outcome measures for opioids prescriptions were (1) the rate of patients with a prescription per 10 000 patients, (2) the quarterly mean days’ supply per prescription, and (3) the quarterly mean number of prescriptions per patient.

**Results:**

The study included a mean (SD) of 3.05 (0.86) million patients annually across the US (mean [SD] age, 43.7 [9.6] years; mean [SD] 59.0% [0.32%] female). Medical cannabis dispensary openings were associated with significant reductions in all opioid outcomes. The rate of patients with cancer with opioid prescriptions changed by −41.07 per 10 000 (95% CI, −54.78 to −27.36 per 10 000; *P* < .001), the quarterly mean days’ supply by −2.54 days (95% CI, −3.16 to −1.92 days; *P* < .001), and the mean number of prescriptions per patient by −0.099 (95% CI, −0.121 to −0.077; *P* < .001). Recreational dispensary openings were also associated with reductions in opioid outcomes, though estimated treatment effects were smaller. The rate of prescriptions changed by −20.63 per 10 000 (95% CI, −35.35 to −5.91 per 10 000; *P* = .049), the mean daily supply by −1.09 days supplied per prescription (95% CI, −1.72 to −0.46 days; *P* = .04), and the mean number of prescriptions per patient by −0.097 (95% CI, −0.134 to −0.060; *P* = .01).

**Conclusions and Relevance:**

This study’s findings indicate cannabis may be a substitute for opioids in the management of cancer-related pain. However, further research directly observing cannabis use is needed to evaluate the efficacy of cannabis as a treatment for cancer-related pain.

## Key Points

**Question:**

Is cannabis availability through medical or recreational cannabis dispensary openings associated with opioid prescription use among commercially insured patients diagnosed with cancer in the US?

**Findings:**

In this repeated cross-sectional study following a mean of 3.05 million patients annually, significant reductions were found in the rate of patients with cancer with opioid prescriptions, the mean daily supply, and the mean number of prescriptions per patient after medical and recreational cannabis dispensary openings.

**Meaning:**

These findings indicate that medical or recreational cannabis laws may be significantly associated with reduced opioid use among patients diagnosed with cancer.

## Teaser

This cross-sectional study examines the association of medical and recreational cannabis dispensary availability with prescription opioid dispensing among commercially insured patients with cancer in the US.

## Introduction

Pain is a prevalent cancer-associated symptom reported by more than 65% of patients with advanced cancer.^1^ Cannabis has shown potential in treating cancer-related pain and alleviating adverse effects from cancer treatments.^2,3,4^ To date, 39 states and Washington, DC, have enacted medical cannabis laws (MCLs) providing cannabis availability for patients with qualifying conditions, including cancer, while 24 states and Washington, DC, have passed recreational cannabis laws (RCLs) legalizing adult-use cannabis. While opioids remain the recommended treatment for cancer pain,^5,6^ these patients may benefit from cannabis availability for adjuvant therapy. Further, cannabis use may reduce opioid use more among patients with cancer whose pain is not well managed with opioids or who experience negative effects of opioid use.

Although existing literature finds MCLs are associated with improved pain-related outcomes among patients with newly diagnosed cancer,^2^ no studies examine the effects of RCLs on opioid use among populations with cancer diagnoses or of any cannabis availability on opioid use across subpopulations of patients who may have been traditionally undertreated for pain.^7,8^ This study fills these gaps by implementing a synthetic control design accounting for variation between populations in states with and without medical cannabis dispensaries (MCDs) and recreational cannabis dispensaries (RCDs).

## Methods

### Data and Measures

We extracted data from Optum’s deidentified Clinformatics Data Mart database (CDM) on patients aged 18 to 64 years with an opioid prescription dispensed between January 1, 2007, and December 31, 2020. Clinformatics includes claims data for approximately 20% of the commercially insured US adult population, representing 15 to 20 million individuals annually. CDM includes information regarding sex, age, race and ethnicity (categorized as Asian, Black, Hispanic, or White based on classifications available in the claims database), and cancer diagnoses. We included patients with cancer with at least 6 months of continuous insurance enrollment during the study period. This cross-sectional study followed the STROBE reporting guideline. The University of Georgia institutional review board approved this study and waived the need for informed consent given the use of deidentified data.

### Outcome and Exposure Variables

We constructed 3 outcome measures of opioid dispensing at the state-quarter level: number of opioid prescriptions filled per 10 000 enrollees with cancer, mean days’ supply per prescription, and mean number of prescriptions per patient. To leverage statistical power, we included other medications as placebos. We also stratified outcomes by age, sex, and race and ethnicity because subpopulations of patients including women and racial and ethnic minorities have historically experienced disparate treatment of pain.^7,8^

We used 2 policy measures as exposure variables: (1) whether a state had open MCDs and (2) whether a state had open RCDs. Data on dispensary openings follow previous studies.^9^ Supplementary analyses use MCL and RCL implementation dates.

### Statistical Analysis

Data were analyzed between December 2024 and February 2025. We used a synthetic control method to compare states with legal cannabis availability through dispensaries to states without availability.^9^ In this method, a synthetic control series is constructed from a weighted mean of the outcome series from never-treated states. Donor units’ outcome series are weighed to match the pretreatment outcome variable series for each treated unit before cannabis availability. The forecasting matching weights from the preintervention period is used to project the counterfactual series of a treated state.^10,11^ Treatment effects reflect the difference between the treated states’ actual series and the synthetic control series after the intervention. This method allows us to assess state-level outcomes. For inference, we rely on the estimation of placebo treatment effects, which provide a distribution for a zero-treatment effect. *P* values indicate how many placebo effects are below decreases or above increases in real treatment effects. *P* < .05 indicated significance. Our method accounts for limitations of difference-in-differences designs, including violation of parallel trends and staggered adoption.^10,11^ The eMethods in Supplement 1 provides detailed methodology, and a case study is shown in eFigure 1 in Supplement 1.

## Results

**Figure 1.**
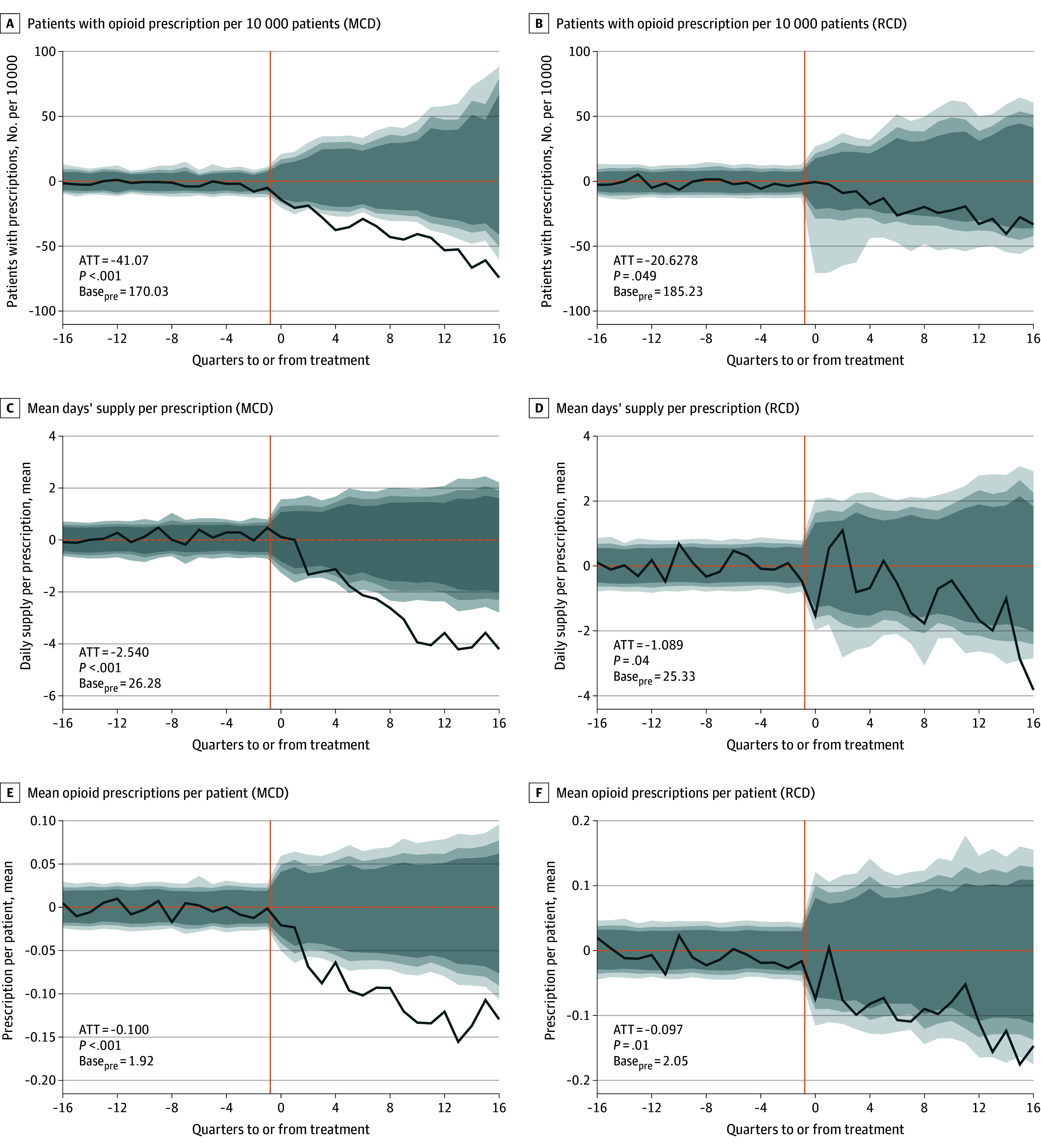
Changes in Opioid Prescription Dispensing (Average Treatment on the Treated) We obtained treatment series from the enrollment-weighted mean across all treated states of the difference between the real observed data with the estimated synthetic control. We approached inference by using an analogous mean across individual series of placebo treatment effects and randomly selecting permutations of treated states to calculate a placebo average effect. We repeated this random draw of series 5000 times. The fading gray areas indicate 90% (light gray), 95% (medium gray), and 99% (dark gray) CIs from the distribution of placebo mean treatments. The solid orange line shows the treatment quarter. ATT indicates average treatment on the treated; Base_pre_, average of the outcome variable the year before MCDs or RCDs opened across treated states; MCD, medical cannabis dispensary; RCD, recreational cannabis dispensary.

The population included a mean (SD) of 3.05 (0.86) million patients (mean SD] age, 43.7 [9.6] years; mean [SD] sex: 59.0% [0.32%] female and 41.0% [0.32%] male; mean [SD] race and ethnicity: 3.7% [0.31%] Asian, 8.0% [0.36%] Black, 9.1% [0.77%] Hispanic, 74.2% [1.38%] White, and 4.9% [2.1%] unknown). Results indicated consistently negative, statistically significant reductions in opioid prescribing after both MCD and RCD openings (Figure 1, Table). MCD openings were associated with reductions in all opioid outcomes. Rate of patients with cancer with opioid prescriptions changed by −41.07 per 10 000 (95% CI, −54.78 to −27.36 per 10 000; *P* < .001), mean days’ supply per prescription changed by −2.54 days (95% CI, −3.16 to −1.92 days; *P* < .001), and mean number of prescriptions per patient changed by −0.099 (95% CI, −0.121 to −0.077; *P* < .001). RCD openings were also associated with reductions in all outcomes. Rate of patients with cancer with opioid prescriptions changed by −20.63 per 10 000 (95% CI, 35.35 to −5.91 per 10 000; *P* < .01), quarterly mean days’ supply changed by −1.09 days per prescription (95% CI, −1.72 to −0.46 days; *P* < .01), and mean number of prescriptions per patient changed by −0.097 (95% CI, −0.134 to −0.060; *P* < .01).

**Table.** Association of Cannabis Dispensary Openings With Opioid Prescription Dispensing Outcomes^a^

Outcome	Data
**Medical cannabis dispensaries**	
Patients with prescription per 10 000 patients, mean (95% CI)	−41.07 (−54.78 to −27.36 )^b^
Base	170.0
Percent change	−24.15%
Daily supply, mean (95% CI), d	−2.54 (−3.16 to −1.92)^b^
Base	26.28
Percent change	−9.67%
No. of prescriptions per patient, mean (95% CI)	−0.099 (−0.121 to −0.077)^b^
Base	1.92
Percent change	−5.17%
**Recreational cannabis dispensaries**	
Patients with prescription per 10 000 patients, mean (95% CI)	−20.63 (−35.35 to −5.91)^c^
Base	185.2
Percent change	−11.14%
Daily supply, mean (95% CI), d	−1.09 (−1.72 to −0.46)^d^
Base	25.33
Percent change	−4.30%
No. of prescriptions per patient, mean (95% CI)	−0.097 (−0.134 to −0.060)^e^
Base	2.050
Percent change	−4.74%

^a^ We obtained treatment series from the enrollment-weighted mean across all treated states of the difference between the real observed data and the estimated synthetic control. We approach inference using an analogous mean across individual series of placebo treatment effects and randomly selecting permutations to calculate a placebo average effect. We repeated this random draw of series 5000 times.

^b^ *P* < .001.

^c^ *P* = .049.

^d^ *P* = .04.

^e^ *P* = .01.

**Figure 2.**
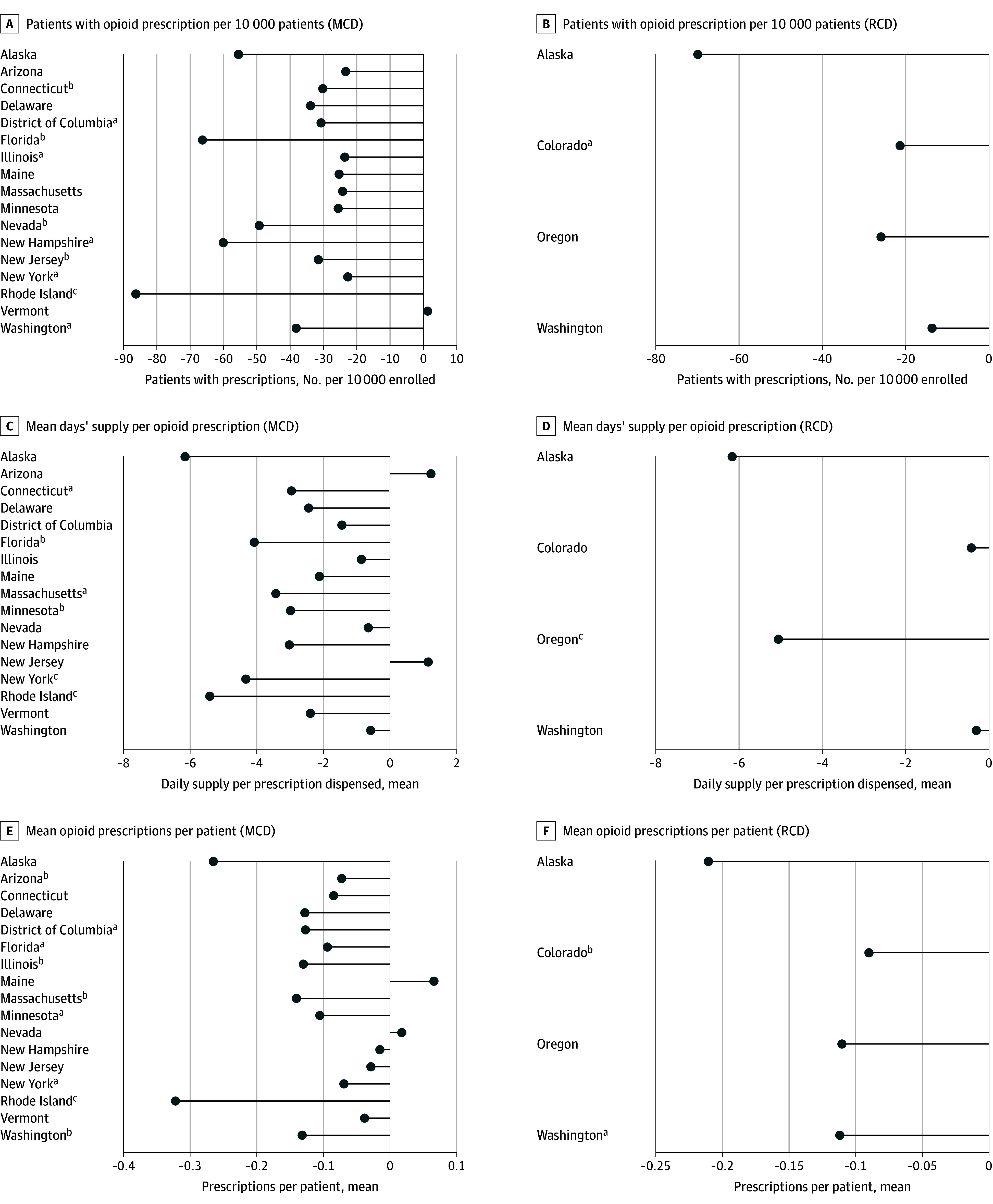
State-Level Treatment Outcomes for Opioid Prescription Dispensing We obtained treatment series from the enrollment-weighted mean across all treated states of the difference between the real observed data with the estimated synthetic control. We approached inference using an analogous mean across individual series of placebo treatment effects and randomly selecting permutations to calculate a placebo average effect. We repeated this random draw of series 5000 times. Outcomes were calculated quarterly for patients with cancer. MCD indicates medical cannabis dispensary; RCD, recreational cannabis dispensary. ^a^*P* < .10. ^b^*P* < .05. ^c^*P* < .01.

Figure 2 illustrates state-level outcomes. In most treated states, we estimated reductions in opioid prescribing outcomes relative to synthetic counterfactuals. We found no significant increases in opioid prescribing outcomes following MCD or RCD openings. We estimated negative associations for all opioid prescribing measures following RCD openings. eTable 1 in Supplement 1 shows state-level coefficients. We did not find significant differences in policy outcomes based on age, sex, or race and ethnicity (eTable 2 in Supplement 1). eFigure 2 and eTable 3 in Supplement 1 show MCL and RCL results. A sensitivity check excluding states that adopted opioid prescribing caps within 2 quarters of MCD or RCD showed unchanged results (eTables 4 and 5 in Supplement 1).

## Discussion

We found significant reductions in all measures of opioid prescription dispensing following MCD and RCD openings. These findings are consistent with prior research suggesting that cannabis may serve as a substitute for opioids in managing pain.^12,13^ Reductions in opioid prescribing rates and daily supply were larger when MCDs were operational than when MCL was implemented, highlighting the potential impact of easier cannabis availability. We estimated smaller reductions in opioid prescribing associated with RCD openings.

Similar to existing literature,^2,12^ we found larger reductions in extensive than intensive measures, which may indicate that patients with lower pain levels are more likely to substitute cannabis for opioids or that prescriber behavior changes following cannabis availability. Prescription drug monitoring programs (PDMPs) allow prescribers in some states to identify patients who use medical cannabis, which may impact prescribing practices.^14^ Regardless, there is imperfect information between prescribers and patients regarding recreational cannabis use. Thus, findings suggesting that recreational cannabis availability is associated with reduced opioid use cannot be attributed solely to prescriber caution. Concurrent opioid prescribing cap laws may also reduce opioid prescribing, although recent evidence suggests these policies did not impact opioid prescribing among patients with noncancer chronic pain.^15^ Furthermore, many PDMP and opioid prescribing cap policies exempt patients with cancer.

We did not find heterogeneous associations across age, sex, or race and ethnicity, indicating that dispensary openings may influence opioid prescription patterns similarly across demographic subpopulations. Findings suggest that cannabis availability may help diverse patients equally manage cancer-related pain if the observed reductions reflect substitution to cannabis.

### Limitations

This study has limitations. First, we observed associations at the state-quarter level, raising concern for ecological fallacy. Second, other state-level policies adopted concurrently with MCD or RCD openings could confound results; however, treated states would have to adopt the same policy at the same time to bias aggregate estimates. Third, the Clinformatics database has heterogeneous coverage across states and includes only commercially insured patients, limiting generalizability to publicly insured populations. We also excluded Medicare-eligible patients 65 years or older. Although cancer is a common diagnosis among older adults, younger adults with cancer diagnoses may pursue aggressive treatments requiring pain management. Fourth, for RCD, we only followed 4 states. Fifth, while the synthetic control method minimizes bias in estimating policy effects, it may not fully capture all relevant factors influencing prescription patterns.

## Conclusions

Results of this study suggest that cannabis may serve as a substitute for opioids in managing cancer-related pain, underscoring the potential of cannabis policies to impact opioid use. Further research should explore individual-level impacts, the mechanisms underlying these changes, and the long-term effects of cannabis policies on cancer pain management.

## Notes

Supplement 1.
eMethods.
**eTable 1. **State-level average treatment on treated: Dispensary openings on opioid prescription dispensing **eTable 2. **Average treatment on treated: Opioid prescription dispensing by demographics **eTable 3. **Average treatment on treated: Cannabis legalization on opioid prescription dispensing **eTable 4. **MCL, RCL, and Opioid prescribing cap policy dates **eTable 5. **Sensitivity check: Dispensary openings on opioid prescription dispensing **eFigure 1. **Example of Synthetic control Estimation **eFigure 2. **Average treatment on the treated: Opioid prescription dispensing

Supplement 2.
Data Sharing Statement
