# Supplementary material for: Cannabis Laws and Opioid Use Among Commercially Insured Patients With Cancer Diagnoses
Source: JAMA Health Forum. 2025 Oct 17;6(10):e253512. doi: 10.1001/jamahealthforum.2025.3512 (PMC12534851; doi:10.1001/jamahealthforum.2025.3512)
Supplement: Supplement 1. — eMethods. eTable 1. State-level average treatment on treated: Dispensary openings on opioid prescription dispensing eTable 2. Average treatment on treated: Opioid prescription dispensing by demographics eTable 3. Average treatment on treated: Cannabis legalization on opioid prescription dispensing eTable 4. MCL, RCL, and Opioid prescribing cap policy dates eTable 5. Sensitivity check: Dispensary openings on opioid prescription dispensing eFigure 1. Example of Synthetic control Estimation eFigure 2. Average treatment on the treated: Opioid prescription dispensing [file jamahealthforum-e253512-s001.pdf]

Supplemental Online Content

Lozano-Rojas F, Bethel V, Gupta S, Steuart SR, Bradford WD, Abraham AJ. Cannabis laws and opioid use among commercially insured patients with cancer diagnoses. *JAMA Health Forum*. 2025;6(10):e253512. doi:10.1001/jamahealthforum.2025.3512

eMethods.

eTable 1. State-level average treatment on treated: Dispensary openings on opioid prescription dispensing

eTable 2. Average treatment on treated: Opioid prescription dispensing by demographics

eTable 3. Average treatment on treated: Cannabis legalization on opioid prescription dispensing

eTable 4. MCL, RCL, and Opioid prescribing cap policy dates

eTable 5. Sensitivity check: Dispensary openings on opioid prescription dispensing

eFigure 1. Example of Synthetic control Estimation

eFigure 2. Average treatment on the treated: Opioid prescription dispensing

This supplemental material has been provided by the authors to give readers additional information about their work.

© 2025 Lozano-Rojas F et al. JAMA Health Forum.

eMethods

1. State Sample Selection

We aim to estimate the average treatment effect of each policy lever in states that have opened cannabis dispensaries (either Medical or Recreational). To ensure a consistent analysis, we define a study window that allows us to track treated states for at least 16 quarters before implementation, the quarter of implementation itself, and at least 16 quarters after—totaling a minimum of 33 quarters. This approach enables us to assess the intermediate-term effects of cannabis policies, addressing a gap in the literature, which has largely focused on the first two years post-implementation. To illustrate our selection process, we provide two examples. California legalized medical cannabis in 1996, placing its implementation date outside our study timeframe. Similarly, Michigan enacted recreational cannabis laws in the last quarter of 2018, limiting post-implementation observation to only eight quarters. As a result, we classify these states as early and late adopters, respectively, and exclude them from our analysis. Ultimately, our study follows 17 for Medical Cannabis with Dispensaries Open and four for Recreational Cannabis with Dispensaries Open.[1] On the other hand, we include all never-treated states as potential donors for synthetic control, that is in the case of medical dispensaries there are 19 states that never had a dispensary on their jurisdiction, and, on the other hand, there are 43 states that have never had a recreational dispensary.

These choices allow us to overcome compositional biases and follows the literature that aims to estimate medium to longer term effects from cannabis legalization, even if we are left with a limited number of states to follow, as in the case of RCL. Other studies exploring RCLs with a limited number of states include: two recreational cannabis states,[2] three to four recreational cannabis states,[3] and five recreational cannabis states.[4] We provide an additional exercise following the time at which the policy became effectively legal as a robustness check, and this results in a larger set of states in the case of recreational cannabis (seven states), with the same concluding take-aways.

2. Synthetic Control Estimation

To examine the association between cannabis access and opioid prescription dispensing, we compared patients with legal cannabis access through dispensaries to patients without legal cannabis access. However, as documented in recent literature, analysis utilizing a difference-in-differences framework may be limited due to staggered MCL adoption across states and limited comparability stemming from violations to the parallel trends assumption.[51] We instead implemented a synthetic control methodology.[1,6,7]

In this method, a donor pool of never-treated states-medications was used to construct a synthetic untreated outcome variable estimate (the counterfactual) to pair with each treated state's outcome series. For each case study (e.g. Florida opioids under MCL), we follow the never-treated state-medication series. Considering that we only have 19 never-treated states under MCL Dispensary, we expand the outcome series to include additional medications. We follow three series for pain treatment (opioid, NSAIDs and other pain) and five series for mental health treatment (antidepressant, antipsychotics, benzodiazepines, z-drugs and barbiturates). We normalize the outcome series in terms of the pre-treatment mean and standard deviation for each case study of both the treated series and all the donors. This expansion is based on a similar common set of assumptions (Hollingsworth & Wing, 2020).

The synthetic control procedure weighs the donor (untreated) units' outcome variable series to match the pretreatment outcome variable series for each treated unit. The aim is to construct a synthetic untreated series that matches as closely as possible to the observed series from the treated unit prior to the policy going into effect. Those same weights are used to construct a synthetic untreated outcome for the time periods when the treated unit is treated; this represents a counterfactual estimate of what would have happened to the outcome in the treated units had the policies not gone into effect.

Thus, if (for example) medical dispensaries are associated with total dispensing rates, any deviation from the synthetic control series represents the treatment effect, relative to the pre-policy baseline.[1,6,8] This allows us to generate an overall treatment effect and a state-specific treatment effect (our case studies). Our methodology accounts for limitations of the standard difference-in-differences design, such as the lack of comparability due to the

© 2025 Lozano-Rojas F et al. JAMA Health Forum.

violation of the parallel trends assumption, or staggered adoption treatment timing.[8,9] We are interested in estimating a treatment effect for each state with a dispensary (α_{it}) as in the equation below:

$$Y_{it} = Y_{it}^{N} + \alpha_{it} . D_t \ \ where\ D_t = \begin{cases} 1\ if\ i = 1\ and\ t \geq 0 \\ 0 \qquad otherwise \end{cases}$$

Where, in the context of potential outcomes notation, Y_{it}^{N}, is the value of the prescription fill in the absence of treatment, and the treatment effect is α_{it} in state-medication and period observations with recreational cannabis policies (when $D_{it} = 1$). Y_{it}^{N} is always observed for the never treated states. It is observed for treated states as well, but only in periods prior to the implementation of recreational cannabis policies. We estimate Y_{it}^{N} for treated states after treatment (separately for each policy lever), by estimating separate state-level case studies. In each case study, the treated state-medication is denoted by $i = 1$, as the remainder of the treated units are not considered jointly, such that in synthetic control estimation there is only a treated state-medication series. The counterfactual outcome of interest is denoted by $\widehat{Y_{it}^{N}}$, and we estimate this term using the ADH synthetic control method with the pool of donor units ($i \geq 2$). The synthetic control estimation procedure generates a set of weights $(w_2^*, \ldots, w_J^*)$, which are used to aggregate the contribution of all donor units to generate the "synthetic" control hat ($\widehat{Y_{it}^{N}}$). This synthetic series, $\widehat{Y_{it}^{N}}$, is constructed out of a selection of weights such that $\widehat{Y_{it}^{N}}$ approximates, as closely as possible, the treated series when $Y_{it} = Y_{it}^{N}$, in the pre-policy period ($t < 0$). We rely exclusively on pre-intervention outcomes to calibrate the synthetic control model. This means that we use lags of in the pre-treatment as covariates.

The estimated synthetic control ($\widehat{Y_{it}^{N}}$), is then projected into the post-policy period and used as the counterfactual outcome against which the treated state's observed outcome is compared. The difference between the two is interpreted as the treatment effect of the policy in period t, represented as:

$$\widehat{\alpha_{1t}} = Y_{1t} - \widehat{Y_{1t}^{N}}\text{, where } \widehat{Y_{1t}^{N}} = \sum_{i=2}^{J} w_i^* \cdot Y_{it}$$

The average treatment effect is obtained by calculating the simple average difference between the treatment and synthetic control across all the post-period units. This is the

$$\overline{\alpha_1} = \frac{1}{17} \cdot \sum_{t=0}^{T=16} \left(Y_{1t} - \widehat{Y_{1t}^{N}} \right) = \Sigma \left(\frac{\widehat{\alpha_{1t}}}{17} \right)$$

We present the average treatment effect on the treated (ATT) states using a permutation inference.[10,11]Thus, we are able to assess which states are responsible for the overall association and whether there is any meaningful state heterogeneity. Finally, we perform a cross-fitting validation to account for overfitting (e.g., a very sharp match of the synthetic control in the training data from the pre-policy period that biases the forecasts in the post period).[6,12] Our findings are robust to this form of misspecification. We obtain the series of the estimated synthetic control using the *synth* command in Stata 18. This command relies on the algorithm developed by Abadie, Diamond and Hainmueller (2010, 2015).

To illustrate our implementation, we present a case study in eFigure 1. Panel A shows New Jersey's observed trend in opioid prescriptions per 10,000 enrollees (black line) alongside its synthetic control estimate (gray line). A noticeable gap emerges post-policy, suggesting a treatment effect. Panel B identifies the positively weighted donor series used to construct the synthetic control, drawn from a diverse set of states and medications. Panel C presents the estimated treatment effect for New Jersey, placebo effects from other donor units, and confidence intervals at the 90% and 95% levels. New Jersey's treatment effect is consistently more negative than most placebo effects. We use 149 donor series from 19 never-treated states and eight medication types, excluding three without pre-treatment variation. For inference, we include only placebo series meeting a pre-treatment balance threshold, a Cohen's D < 0.25, where Cohen's D is defined as the standardized difference between the real and synthetic means in the pre-intervention period. For New Jersey, no placebo fails this criterion, so the treatment effect is evaluated against 149 valid placebo series. We rank the treatment effects and obtain one-tail p-values by calculating the share of placebo treatments that are below (above) negative (positive) treatment effects.

© 2025 Lozano-Rojas F et al. JAMA Health Forum.

Following the implementation of the MCL dispensary policy in New Jersey, we estimate an average reduction of 31.4 patients with opioid prescriptions per 10,000 enrollees ($p = 0.0403$), which represents a 20.1% decline from the pre-policy baseline. Out of 149 placebo treatment effects six fall below the one we find for New Jersey ($p = 6/149 = 0.0403$). Individual treatment effects for all treated states are reported in Figure 2 of the main manuscript and in eTable 1. We repeat this process for each treated unit to obtain the average treatment on the treated weighted by the state average number of enrollees. Inference for the ATT is conducted by comparing the real ATT to different placebo ATTs. In the case of MCL Dispensary, we take 5,000 draws from feasible combinations of the 149-donor series into the 17 treated ones to build each placebo ATT. We proceed ranking and comparing in the same described fashion. For instance, the ATT reported in Panel A of Figure 1 for opioid prescription rates following MCL Dispensary shows a drop of 41 patients with opioid prescriptions, and it has a p-value of 0.000. This means that the real effect was smaller than the 5,000 placebo ATTs obtained for inference.

3. Medical and Recreational Cannabis Legalization

We conduct analyses using the date that medical and recreational cannabis became legal (MCL and RCL) as alternative treatment timing to the date that medical cannabis dispensaries or recreational cannabis dispensaries opened. MCD openings were associated with larger reductions in opioid prescribing than MCL (Table 1/eFigure 2/eTable 2). Reductions in opioid prescribing associated with RDC openings were slightly smaller in magnitude than reductions associated with MCD openings or reductions associated with RCL (Figure 1/eFigure 2/eTable 2).

4. State-level analyses

Medical cannabis dispensary openings are associated almost uniformly with declines in all measures of opioid dispensing across states. Only Vermont and Maine show small, insignificant increases in outcomes. Following medical cannabis dispensary openings in Vermont, the rate of opioid prescriptions per 10,000 enrollees increased by 1.3%, and in Maine, the average number of prescriptions per patient increased by 3.1%. The magnitude of estimated reductions varies across states. Treated states in the top 50th percentile for percent reduction in all three opioid dispensing outcomes include Alaska, Delaware, Washington, D.C., Florida, and Rhode Island. Arizona, Iowa, and Ohio are states in the lowest 50th percentile of estimated percent reductions across all outcomes. With the exception of Alaska, Delaware, Maine, and Vermont, the estimated reductions in at least one opioid dispensing outcome following medical cannabis dispensary openings is statistically significant. The associated reductions are statistically significant for all outcomes in Florida and Rhode Island.

Recreational cannabis dispensary openings are also consistently and exclusively associated with reductions in opioid dispensing outcomes. However, effects are generally smaller, and we only estimate statistically significant reductions in any of the three opioid dispensing outcomes in Washington, Oregon, and Colorado following recreational cannabis dispensary openings.

5. Sensitivity Check

Although the majority of PDMP and opioid prescribing cap policies do not apply to patients with cancer, we test whether the exclusion of states that implemented opioid prescribing cap policies within two quarters of MCD/RCD adoption affects our treatment effects. The treatment effects displayed in eTable 5 are the average of state-level treatment effects excluding states that implemented opioid prescribing caps within two months of dispensary openings. For medical cannabis analyses, we exclude New York when averaging state-level treatment effects. Recreational cannabis analyses are not affected. Results are nearly unchanged.

© 2025 Lozano-Rojas F et al. JAMA Health Forum.

6. References

© 2025 Lozano-Rojas F et al. JAMA Health Forum.

1. Bradford AC, Lozano-Rojas F, Shone HB, Bradford WD, Abraham AJ. Cannabis laws and utilization of medications for the treatment of mental health disorders. JAMA Netw Open. 2024 Sep;7(9):e2432021.
2. Dave D, Liang Y, Muratori C, Sabia J. The Effects of Recreational Marijuana Legalization on Employment and Earnings. Cambridge, MA; 2022 Dec.
3. Jiang S, Miller K. Watching the grass grow: does recreational cannabis legalization affect retail and agricultural wages? J Cannabis Res. 2022 Dec 26;4(1):42.
4. Hollingsworth A, Wing C, Bradford AC. Comparative Effects of Recreational and Medical Marijuana Laws on Drug Use among Adults and Adolescents. J Law Econ. 2022 Aug 1;65(3):515–54.
5. Roth J, Sant'Anna PHC, Bilinski A, Poe J. What's trending in difference-in-differences? A synthesis of the recent econometrics literature. J Econom. 2023 Aug;235(2):2218–44.
6. Abadie A. Using synthetic controls: Feasibility, data requirements, and methodological aspects. J Econ Lit. 2021 Jun;59(2):391–425.
7. Abadie A, Diamond A, Hainmueller J. Synthetic Control Methods for Comparative Case Studies: Estimating the Effect of California's Tobacco Control Program. J Am Stat Assoc. 2010 Jun;105(490):493–505.
8. Hollingsworth A, Wing C. Tactics for design and inference in synthetic control studies: An applied example using high-dimensional data. SSRN Electron J. 2020;
9. Callaway B, Sant'Anna PHC. Difference-in-Differences with multiple time periods. J Econom. 2021 Dec;225(2):200–30.
10. Cavallo E, Galiani S, Noy I, Pantano J. Catastrophic natural disasters and economic growth. Rev Econ Stat. 2013 Dec;95(5):1549–61.
11. Hagemann A. Placebo inference on treatment effects when the number of clusters is small. J Econom. 2019 Nov;213(1):190–209.
12. Chernozhukov V, Wuthrich K, Zhu Y. A t-test for synthetic controls. arXiv [econEM]. 2018 Dec;
13. McCourt AD, Tormohlen KN, Schmid I, Stone EM, Stuart EA, Davis CS, et al. Effects of Opioid Prescribing Cap Laws on Opioid and Other Pain Treatments Among Persons with Chronic Pain. J Gen Intern Med. 2023 Mar 1;38(4):929–37.

© 2025 Lozano-Rojas F et al. JAMA Health Forum.

eTable 1: State-level average treatment on treated: Dispensary openings on opioid prescription dispensing

	Patient Prescription Dispensing Rate		Daily Supply per Prescription		Average Prescriptions per Patient	
**State**	**MCL Legal**	**MCL Dispensary**	**MCL Legal**	**MCL Dispensary**	**MCL Legal**	**MCL Dispensary**
**AK**		-55.497		-6.142		-0.264
**(Std. Dev.)**		(81.362)		(13.833)		(0.613)
**Baseline**		244.21		31.07		2.135
**% Change**		-22.73		-19.77		-12.39
**AZ**		-23.170		1.224		-0.072**
		(22.372)		(1.226)		(0.027)
		231.23		29.17		2.092
		-10.02		4.19		-3.46
**AR**	0.755		-3.252*		-0.095	
	(55.299)		(2.263)		(0.118)	
	230.18		29.21		2.009	
	0.33		-11.14		-4.73	
**CT**	-13.679	-30.140**	0.332	-2.958*	-0.003	-0.085
	(17.027)	(17.366)	(1.492)	(1.969)	(0.100)	(0.092)
	164.95	149.91	25.34	26.98	1.995	2.035
	-8.29	-20.11	1.31	-10.96	-0.16	-4.17
**DE**	-57.119*	-33.794	2.054	-2.449	0.060	-0.128
	(40.218)	(47.241)	(4.077)	(5.311)	(0.199)	(0.200)
	216.36	165.72	32.34	31.75	2.207	2.136
	-26.40	-20.39	6.35	-7.71	2.71	-5.98
**DC**		-30.696*		-1.447		-0.127*
		(20.347)		(1.443)		(0.095)
		128.27		17.83		1.657
		-23.93		-8.12		-7.65
**FL**		-66.326**		-4.071**		-0.094*
		(41.185)		(1.632)		(0.050)
		201.31		30.11		1.908
		-32.95		-13.52		-4.92
**IL**	-27.158**	-23.459*	-0.268	-0.850	-0.137***	-0.130**
	(15.662)	(18.743)	(1.271)	(1.517)	(0.044)	(0.063)
	171.37	161.64	24.90	24.65	2.013	1.852
	-15.85	-14.51	-1.08	-3.45	-6.81	-7.02
**IA**	-33.870**		-2.156*		-0.093*	
	(19.970)		(1.367)		(0.080)	
	186.43		27.36		2.083	
	-18.17		-7.88		-4.46	
**LA**	-66.866***		-0.945		-0.097	
	(28.032)		(1.899)		(0.081)	
	269.67		28.86		2.001	
	-24.80		-3.27		-4.87	
**ME**		-25.236		-2.118		0.065
		(27.169)		(7.805)		(0.317)
		185.43		28.87		2.138
		-13.61		-7.34		3.06
	**Patient Prescription Dispensing Rate**		**Daily Supply per Prescription**		**Average Prescriptions per Patient**	

© 2025 Lozano-Rojas F et al. JAMA Health Forum.

State	MCL Legal	MCL Dispensary	MCL Legal	MCL Dispensary	MCL Legal	MCL Dispensary
MA	-28.634*	-24.140	-0.848	-3.429*	-0.067	-0.140**
(Std. Dev.)	(20.028)	(23.668)	(1.422)	(2.071)	(0.074)	(0.065)
Baseline	157.69	123.42	23.81	23.06	1.922	1.832
% Change	-18.16	-19.56	-3.56	-14.87	-3.47	-7.64
MN	-48.286**	-25.497	-2.279**	-2.986**	-0.101*	-0.105*
	(24.849)	(26.973)	(1.221)	(1.250)	(0.063)	(0.070)
	160.01	126.32	24.10	22.40	2.159	2.062
	-30.18	-20.18	-9.46	-13.33	-4.70	-5.08
NV		-49.240*		-0.653		0.018
		(35.035)		(2.793)		(0.134)
		198.60		31.20		1.939
		-24.79		-2.09		0.91
NH	-37.528	-60.058*	4.961**	-2.987	0.021	-0.015
	(29.946)	(48.802)	(2.225)	(4.018)	(0.149)	(0.154)
	160.31	118.16	18.53	25.55	1.890	1.970
	-23.41	-50.83	26.77	-11.69	1.12	-0.76
NJ		-31.422**		1.143		-0.028
		(17.208)		(1.209)		(0.057)
		156.61		23.02		1.876
		-20.06		4.97		-1.50
NY	-30.443**	-22.660	-1.755**	-4.337***	-0.033	-0.069*
	(14.637)	(20.798)	(0.822)	(1.104)	(0.046)	(0.045)
	117.72	105.31	20.65	21.83	1.613	1.629
	-25.86	-21.52	-8.50	-19.87	-2.07	-4.25
OH	-37.406		-0.451		-0.036	
	(36.841)		(1.804)		(0.077)	
	193.77		27.40		1.927	
	-19.30		-1.65		-1.86	
PA	-26.324		-1.789		-0.113	
	(34.012)		(2.101)		(0.093)	
	183.92		28.95		1.970	
	-14.31		-6.18		-5.73	
RI		-86.261***		-5.407***		-0.322***
		(18.609)		(1.186)		(0.064)
		205.82		29.49		2.214
		-41.91		-18.33		-14.53
VT		1.311		-2.393		-0.038
		(41.825)		(5.698)		(0.456)
		97.39		21.69		2.000
		1.35		-11.03		-1.90
WA		-38.194*		-0.587		-0.132**
		(27.372)		(0.945)		(0.068)
		190.65		24.64		2.045
		-20.03		-2.38		-6.45

© 2025 Lozano-Rojas F et al. JAMA Health Forum.

State	Patient Prescription Dispensing Rate		Daily Supply per Prescription		Average Prescriptions per Patient	
	RCL Legal	RCL Dispensary	RCL Legal	RCL Dispensary	RCL Legal	RCL Dispensary
**AK**	-41.743	-69.868	-4.186	-6.172	-0.226	-0.210
**(Std. Dev.)**	(52.540)	(89.019)	(9.393)	(13.145)	(0.568)	(0.597)
**Baseline**	204.42	244.21	38.09	31.07	2.579	2.135
**% Change**	-20.42	-28.61	-10.99	-19.87	-8.78	-9.85
**CA**	-25.617		-3.687**		-0.092*	
	(31.902)		(1.658)		(0.068)	
	151.16		27.61		1.848	
	-16.95		-13.36		-4.98	
**CO**	-22.622*	-21.156*	-0.471	-0.416	-0.070*	-0.089**
	(16.198)	(18.104)	(0.786)	(0.793)	(0.044)	(0.045)
	202.16	186.63	25.47	25.48	2.080	2.062
	-11.19	-11.34	-1.85	-1.63	-3.35	-4.34
**DC**	-18.476		-1.902		-0.100	
	(22.089)		(1.782)		(0.113)	
	114.02		15.55		1.529	
	-16.20		-12.23		-6.56	
**MA**	-10.815		-3.599*		-0.034	
	(42.532)		(2.642)		(0.078)	
	122.31		23.25		1.842	
	-8.84		-15.48		-1.85	
**OR**	-35.209	-25.854	-4.971***	-5.053***	-0.111	-0.110
	(33.605)	(35.896)	(1.523)	(1.709)	(0.111)	(0.117)
	175.19	169.34	26.24	25.35	2.031	2.002
	-20.10	-15.27	-18.94	-19.93	-5.45	-5.48
**WA**	-13.263	-13.522	-0.187	-0.313	-0.111*	-0.111*
	(27.691)	(27.724)	(1.083)	(1.091)	(0.071)	(0.070)
	190.65	190.65	24.64	24.64	2.045	2.045
	-6.96	-7.09	-0.76	-1.27	-5.45	-5.45

© 2025 Lozano-Rojas F et al. JAMA Health Forum.

eTable 2: Average treatment on treated: Opioid prescription dispensing by demographics

Medical Cannabis Dispensaries Open										
Outcomes	All Cancer Patients	Subpopulations with Cancer								
		Sex		Age Group			Race/Ethnicity			
		Female	Male	18-26	27-40	41-64	Black	Asian	Hispanic	White
**Rx pts/10,000**	-41.07***	-37.3***	-30.21***	-14.19**	-22.13**	-37.0***	-42.67***	-21.63***	-25.61***	-42.73***
(s.d.)	(13.71)	(15.69)	(12.52)	(10.07)	(12.28)	(15.78)	(18.48)	(6.52)	(11.91)	(12.32)
% Change	-24.15%	-19.99%	-20.64%	-21.29%	-19.01%	-18.29%	-20.94%	-19.94%	-17.21%	-24.61%
Base	170.0	186.6	146.4	66.6	116.4	202.2	203.8	108.5	148.8	173.7
**Daily supply**	-2.54***	-2.45***	-2.8***	-2.08***	-2.79***	-2.41***	-3.3***	-1.77**	-2.08***	-2.64***
(s.d.)	-0.62	-0.52	-0.72	-0.68	-0.60	-0.63	-1.20	-0.99	-0.72	-0.66
% Change	-9.67%	-9.97%	-9.52%	-17.72%	-16.40%	-8.35%	-13.78%	-10.88%	-10.29%	-9.62%
Base	26.28	24.54	29.42	11.74	16.99	28.91	23.97	16.30	20.26	27.49
**Avg. rx/pt**	-0.099***	-0.113***	-0.114***	-0.114***	-0.139***	-0.106***	-0.123***	-0.083***	-0.121***	-0.1***
(s.d.)	-0.022	-0.021	-0.027	-0.035	-0.028	-0.023	-0.039	-0.039	-0.024	-0.023
% Change	-5.17%	-6.08%	-5.67%	-7.62%	-8.25%	-5.33%	-6.82%	-5.35%	-7.32%	-5.09%
Base	1.919	1.865	2.017	1.499	1.682	1.991	1.805	1.542	1.656	1.972
**Recreational Cannabis Dispensaries Open**										
Outcomes	All Cancer Patients	Subpopulations with Cancer								
		Sex		Age Group			Race/Ethnicity			
		Female	Male	18-26	27-40	41-64	Black	Asian	Hispanic	White
**Rx pts/10,000**	-20.63**	-32.17**	-21.93**	-19.08*	-28.67***	-27.84**	-43.6**	-28.79**	-18.57	-20.79*
(s.d.)	(14.72)	(16.20)	(12.79)	(12.08)	(11.60)	(16.75)	(27.65)	(15.46)	(16.51)	(15.86)
% Change	-11.14%	-15.37%	-14.52%	-22.55%	-21.01%	-12.97%	-21.25%	-22.88%	-10.43%	-11.96%
Base	185.2	209.3	151.0	84.6	136.5	214.6	205.1	125.9	178.0	173.9
**Daily supply**	-1.09**	-0.98*	-1.32	1.07	-1.28*	-1.23**	-4.02*	0.58	-1.92	-1.82**
(s.d.)	(0.63)	(0.72)	(1.40)	(2.29)	(1.02)	(0.76)	(2.92)	(1.53)	(1.74)	(0.90)
% Change	-4.30%	-4.10%	-4.71%	9.43%	-7.36%	-4.41%	-18.65%	3.74%	-7.66%	-6.68%
Base	25.33	24.00	27.96	11.38	17.40	27.81	21.54	15.55	25.11	27.24
**Avg. rx/pt**	-0.097**	-0.108**	-0.091*	0.019	-0.105*	-0.16***	-0.149		-0.163**	-0.054
(s.d.)	(0.037)	(0.044)	(0.065)	(0.119)	(0.067)	(0.038)	(0.136)		(0.078)	(0.086)
% Change	-4.74%	-5.36%	-4.26%	1.12%	-5.61%	-7.58%		-7.35%	-7.97%	-3.28%
Base	2.050	2.010	2.130	1.663	1.870	2.110		2.029	2.050	1.651

© 2025 Lozano-Rojas F et al. JAMA Health Forum.

eTable 3: Average treatment on treated: Cannabis legalization on opioid prescription dispensing

Medical Cannabis Laws	
**Outcome**	**Legal**
**Patients with prescription/10,000 patients**	-35.91***
(s.d.)	(10.08)
Base	174.50
% Change	-20.58%
**Average daily supply**	-1.19**
(s.d.)	(0.56)
Base	25.28
% Change	-4.72%
**Average prescriptions/patient**	-0.075***
(s.d.)	(0.024)
Base	1.95
% Change	-3.86%
**Recreational Cannabis Laws**	
**Outcome**	**Legal**
**Patients with prescription/10,000 patients**	-23.27*
(s.d.)	(16.94)
Base	165.6
% Change	-14.05%
**Average daily supply**	-2.64***
(s.d.)	(0.92)
Base	26.20
% Change	-10.06%
**Average prescriptions/patient**	-0.085**
(s.d.)	(0.039)
Base	1.926
% Change	-4.43%

© 2025 Lozano-Rojas F et al. JAMA Health Forum.

eTable 4: MCL, RCL, and Opioid prescribing cap policy dates

State	MCD	RCD	Opioid Prescribing Cap
**Alaska**	Q4 2016	Q4 2016	Q3 2017
**Arizona**	Q4 2012		Q2 2018
**Connecticut**	Q3 2014		Q3 2016
**Delaware**	Q2 2015		Q2 2017
**Florida**	Q3 2016		Q3 2018
**Illinois**	Q4 2015		
**Maine**	Q2 2011	Q4 2020	Q1 2017
**Massachusetts**	Q2 2015	Q4 2018	
**Minnesota**	Q3 2015		Q4 2019
**Nevada**	Q3 2015		Q1 2018
**New Hampshire**	Q2 2016		Q1 2017
**New Jersey**	Q4 2012		Q2 2017
**New York**	Q1 2016		Q3 2016
**Rhode Island**	Q2 2013		Q1 2017
**Vermont**	Q2 2013		Q3 2017
**Washington**	Q2 2009	Q3 2014	Q1 2019
**Washington, D.C.**	Q3 2013		

Notes: Cells shaded gray implemented opioid prescribing caps within 2 quarters of our primary MCL/RCL dispensary opening treatment measures. New York opened medical cannabis dispensaries and implemented opioid prescribing cap policies within 2 quarters. No states opened recreational cannabis dispensaries within 2 quarters of the opioid prescribing cap. Policy date information about opioid prescribing caps taken from McCourt and coauthors.[13]

© 2025 Lozano-Rojas F et al. JAMA Health Forum.

eTable 5: Sensitivity check: Dispensary openings on opioid prescription dispensing

	**MCL Dispensary**	
**Outcome**	**Without states with caps**	**Regular results**
**Patients with prescription/10,000 patients**	-43.59***	-41.07***
(s.d.)	(15.34)	(13.71)
Base	178.91	170
% Change	-24.36%	-24.15%
**Average daily supply**	-2.29***	-2.54***
(s.d.)	(0.686)	(0.620)
Base	26.89	26.28
% Change	-8.52%	-9.67%
**Average prescriptions/patient**	-0.080**	-0.099***
(s.d.)	(0.028)	(0.022)
Base	2.02	1.92
% Change	-3.96%	-5.17%

Notes: No state from our timeframe had a concomitant RCL and an Opioid Prescription Cap. See eTable 4 for the list of states with both CLs and Opioid prescribing caps.

© 2025 Lozano-Rojas F et al. JAMA Health Forum.

eFigure 1: Example of Synthetic control Estimation

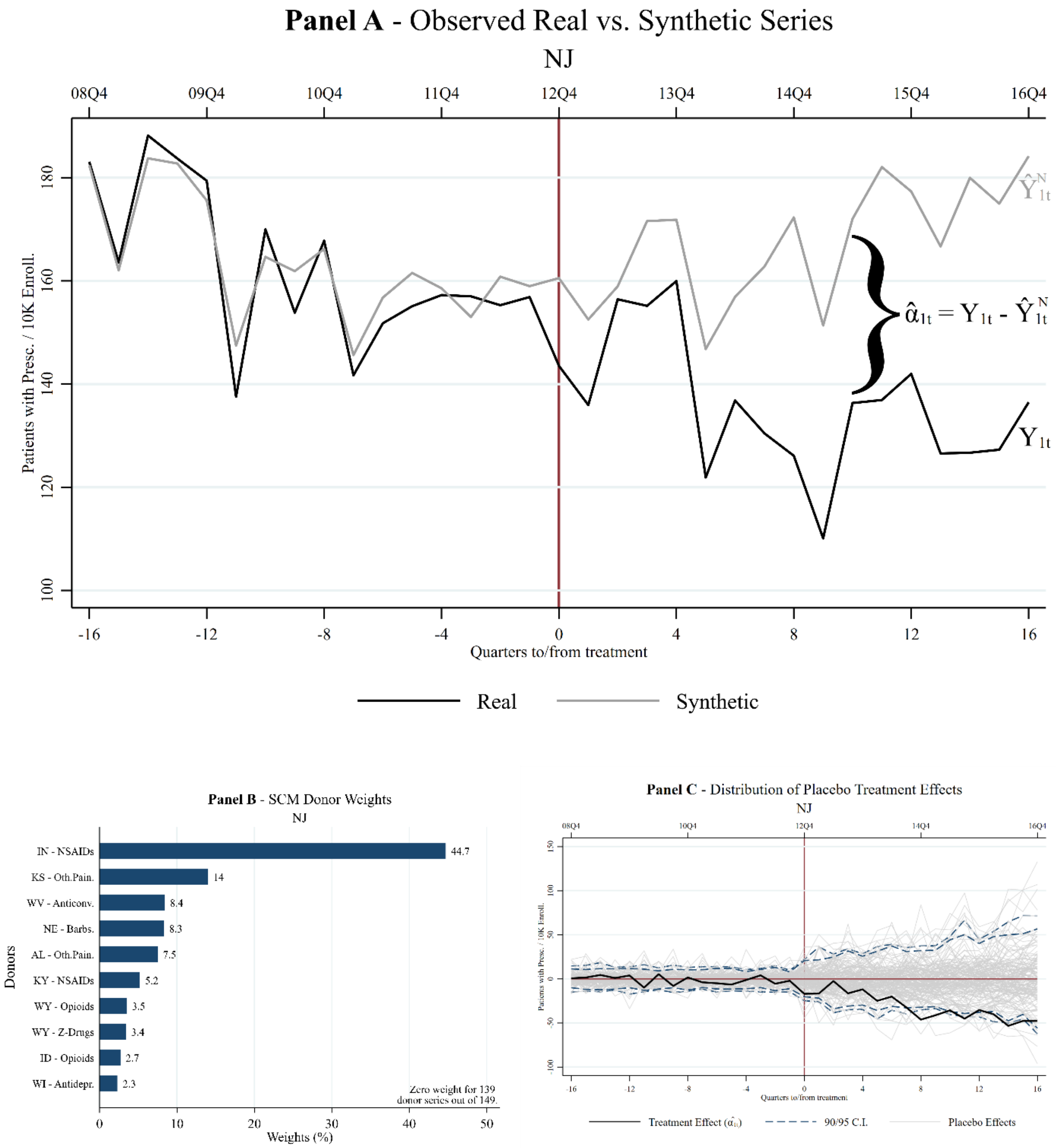

© 2025 Lozano-Rojas F et al. JAMA Health Forum.

eFigure 2: Average treatment on the treated: Opioid prescription dispensing

Panel A – Rate of patients with prescriptions dispensed per 10,000 patients

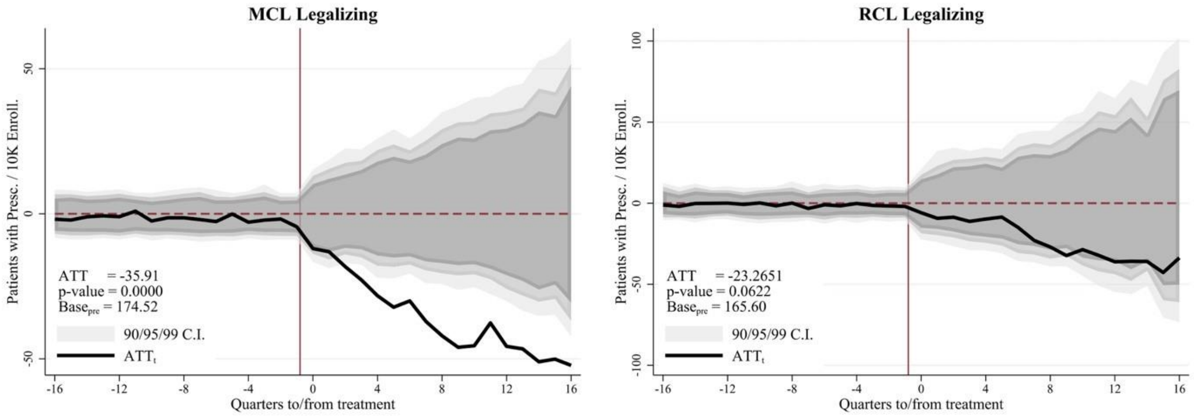

Panel B – Average daily supply per prescription dispensed

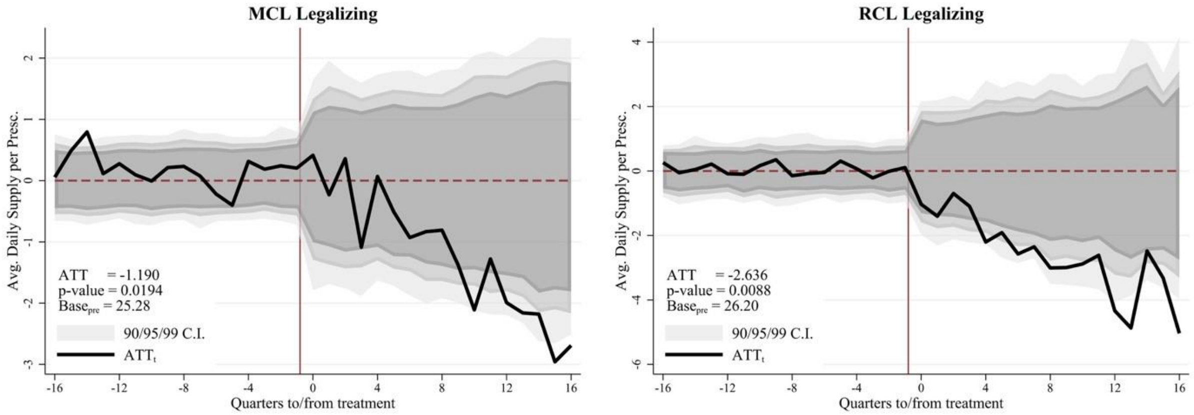

Panel C – Average number of prescriptions dispensed per patient

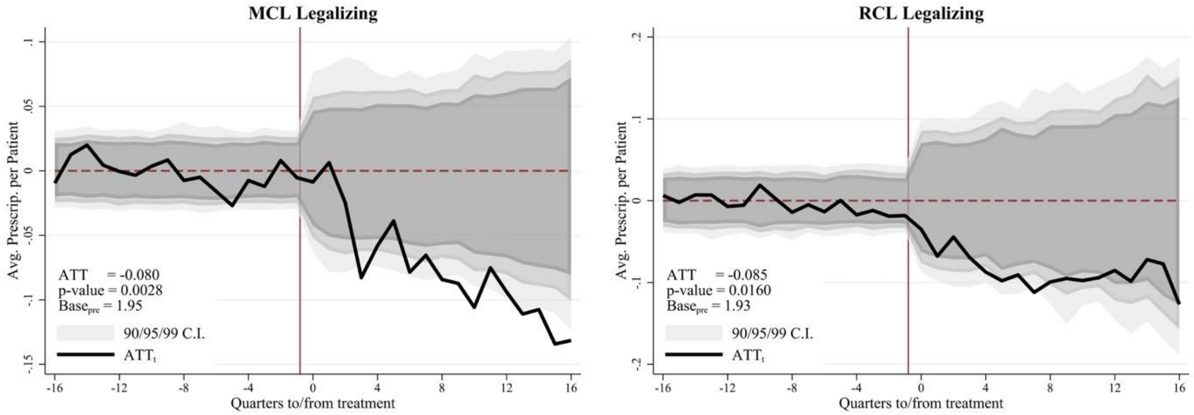

Notes: Outcomes are calculated using information from Clinformatics® aggregated at the state quarter level. We obtain Treatment series from the enrollment-weighted average across all treated states of the difference between the real observed data with the estimated synthetic control. We approach inference using an analogous average across individual series of placebo treatment effects and randomly selecting permutations to calculate a placebo average effect. We repeat this random draw of series 5,000 times. The fading gray areas indicate 90, 95 and 99% confidence intervals from the distribution of placebo average treatments. The solid red line shows the treatment quarter. The different panels include the different outcomes.

© 2025 Lozano-Rojas F et al. JAMA Health Forum.
