# Supplementary material for: Cannabis Laws and Opioid Use Among Commercially Insured Patients With Cancer Diagnoses
Source: JAMA Health Forum. 2025 Oct 17;6(10):e253512. doi: 10.1001/jamahealthforum.2025.3512 (PMC12534851; doi:10.1001/jamahealthforum.2025.3512)
Supplement: Supplement 2. — Data Sharing Statement [file jamahealthforum-e253512-s002.pdf]

Data Sharing Statement

Lozano-Rojas. Cannabis Laws and Opioid Use Among Commercially Insured Patients With Cancer Diagnoses. *JAMA Health Forum*. Published October 17, 2025.
doi:10.1001/jamahealthforum.2025.3512

Data
Data available: No

Additional Information
Explanation for why data not available: The data used in this study are drawn from Optum's deidentified Clinformatics® Data Mart Database and are not publicly available due to licensing and data use agreement restrictions. Access to the data requires a separate agreement with Optum. The analytic code is available upon request from the authors, pending appropriate review and data access approval.
